# Bluetongue virus RNA binding protein NS2 is a modulator of viral replication and assembly

**DOI:** 10.1186/1471-2199-8-4

**Published:** 2007-01-22

**Authors:** Alak Kanti Kar, Bishnupriya Bhattacharya, Polly Roy

**Affiliations:** 1 Department of Infectious and Tropical Diseases, London School of Hygiene and Tropical Medicine, Keppel Street, London, WC1E 7HT, UK; 2 Current address: Dana-Farber Cancer Institute, 44 Binney Street, Jimmy Fund 608, Boston, Massachusetts, 02115, USA

## Abstract

**Background:**

Bluetongue virus (BTV) particles consist of seven structural proteins that are organized into two capsids. In addition, BTV also encodes three non-structural (NS) proteins of which protein 2 (NS2) is the RNA binding protein and is also the major component of virus encoded inclusion bodies (VIBs), which are believed to be virus assembly sites. To investigate the contribution of NS2 in virus replication and assembly we have constructed inducible mammalian cell lines expressing full-length NS2. In addition, truncated NS2 fragments were also generated in an attempt to create dominant negative mutants for NS2 function.

**Results:**

Our data revealed that expression of full-length NS2 was sufficient for the formation of inclusion bodies (IBs) that were morphologically similar to the VIBs formed during BTV infection. By using either, individual BTV proteins or infectious virions, we found that while the VP3 of the inner capsid (termed as "core") that surrounds the transcription complex was closely associated with both NS2 IBs and BTV VIBs, the surface core protein VP7 co-localized with NS2 IBs only in the presence of VP3. In contrast to the inner core proteins, the outer capsid protein VP2 was not associated with either IBs or VIBs. Like the core proteins, newly synthesized BTV RNAs also accumulated in VIBs. Unlike full-length NS2, neither the amino-, nor carboxyl-terminal fragments formed complete IB structures and each appeared to interfere in overall virus replication when similarly expressed.

**Conclusion:**

Together, these data demonstrate that NS2 is sufficient and necessary for IB formation and a key player in virus replication and core assembly. Perturbation of NS2 IB formation resulted in reduced virus synthesis and both the N terminal (NS2-1) and C terminal (NS2-2) fragments act as dominant negative mutants of NS2 function.

## Background

In a number of animal and plant viruses the replication and transcription complexes as well as nucleocapsids, assembly intermediates and virions, accumulate in specific virus induced structures within the host cell cytoplasm described as 'virus assembly factories' or 'virus inclusion bodies' (VIBs) [1-6]. Similar to other members of the *Reoviridae *family, orbiviruses use such specific sites for virus replication and the assembly of virion particles. Bluetongue virus (BTV), the prototype orbivirus, enters the host cells via the two outer capsid proteins, its receptor binding protein VP2 and penetration protein VP5 [7-9]. Once the virion is internalized, both outer capsid proteins are lost within the endosome and the core, consisting of two major (VP3 and VP7), three minor enzymatic (VP1, the viral polymerase; VP4, the capping enzyme and VP6, the helicase) proteins and a genome of ten double-stranded RNA (dsRNA) segments, is released into the cytoplasm. The released core becomes very rapidly associated with a matrix that gradually surrounds the particles to form VIBs. As the infection progresses, these VIBs increase both in size and number and appear to be the sites of orbivirus replication and early viral assembly [5,10-12]. This has largely been inferred from electron microscopy data on the localization of incomplete virus particles within VIBs.

Beside the seven structural proteins, BTV also produces four non-structural proteins (NS1, NS2, NS3 and NS3A) in infected cells. Recently it has been reported that NS1, which is produced abundantly in infected cells is involved in viral morphogenesis, and the two smallest proteins, NS3 and NS3A are involved in virus egress [13,14]. The involvement of the 41 kDa NS2 in virus assembly is also gradually being delineated. NS2 is the only BTV protein that undergoes phosphorylation [15,16]. It is a highly charged hydrophilic protein and exists as a 7S multimeric complex [17-22]. NS2 possesses a single-stranded RNA binding activity and also has a nucleotidyl phosphatase activity [23,24]. These observations suggest that NS2 may have a role in the recruitment and/or retention of RNA for replication.

Expression of BTV NS2 in both, insect and mammalian cells, results in the formation of inclusion bodies (IBs) that are indistinguishable from the VIBs found in virus-infected cells, indicating that NS2 is the major component of the VIBs [17,25,26]. To date, the distribution and localization of BTV proteins and RNAs within the infected cell have mainly been studied by electron microscopy [27]. However, the correlation between what is observed and what is occurring at the molecular level is relatively poor. Nevertheless, the presence of the core, nucleic acids and morphogenic intermediates at the periphery of the VIBs has led to the proposal that replication and assembly of BTV take place within this electron dense structure [12]. In this study we provide experiments to confirm, extend and substantiate the role of NS2 in virus assembly and replication. We describe the generation of several inducible mammalian cell lines expressing complete NS2 as well as different fragments of NS2 including a fragment consisting of the major RNA binding domains [19,21,28,29]. Analysis of these stable cell lines have confirmed that NS2 alone induces the formation of IBs similar to VIBs in BTV infected cells and that they are also capable of recruiting viral core proteins and mRNAs. The IB function of NS2 is essential for virus replication and correct trafficking. Infection of cell lines expressing truncated fragments not only interferes with virus replication but also alters the cellular distribution of BTV particles in accordance with this model.

## Results

### Expression of full-length NS2 in mammalian cells is sufficient for IB formation

**Figure 1:**
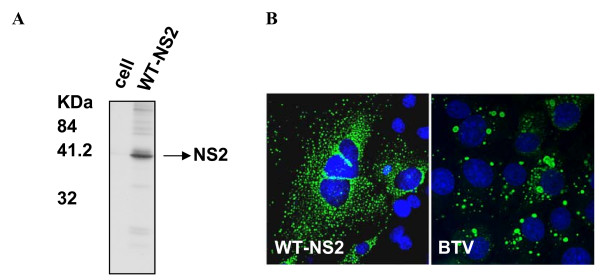
**Full-length NS2 expression by constitutive BSR cells and its intracellular distribution**. (A) Radiolabeled lysate of uninduced (left lane) and induced WT-NS2 cell line (right lane) were immunoprecipited using polyclonal anti-NS2 antibody. The names of the cell lines are given at the top of the panel. The position of NS2 fragment is indicated. (B) Cells expressing NS2 in absence of doxycycline (left) and unmodified BSR cells (right) infected with BTV-10 at 24 h p.i were immunolabeled with an anti-NS2 polyclonal antibody (green). Nuclei were stained with Hoechst 33342.

In order to generate sufficient level of NS2 protein in the absence of other BTV proteins, a mammalian cell line which expresses NS2 has been established using a Tet-off inducible expression system. This is a stringent and tightly controlled gene expression system where the removal of doxycycline from the culture media results in high level transcription of the cloned gene. The full-length NS2 gene was PCR amplified and cloned into pTRE2-Hyg vector, transfected into BSR cells and stable cell lines were selected in the presence of hygromycin as described in the Materials and Methods. Generation of cell lines was confirmed by RT-PCR using specific pairs of primers (data not shown) and ultimately the production of protein was confirmed by radiolabeling and immunoprecipitating the expressed proteins (Fig. 1A).

To examine whether the NS2 expressing cells synthesize structures resembling VIBs, cells were analyzed by immunofluorescence using anti-NS2 antibody (Fig. 1B). The presence of different sizes of globular, dense inclusions was easily detectable in the cells expressing NS2 in the absence of doxycycline. These structures morphologically resemble VIBs in BTV infected BSR cells but the sizes in the latter appear to be larger and comparatively less scattered. The globular structures, in the case of the NS2 expressing cells, were not produced in the presence of 1 μg/ml doxycycline (data not shown), which suppresses NS2 protein expression. These results demonstrate that a continuous mammalian cell line with regulated expression of full-length NS2 protein was constructed.

### Formation of NS2 IBs is not dependent on either cellular microtubule network or intermediate filaments

**Figure 2:**
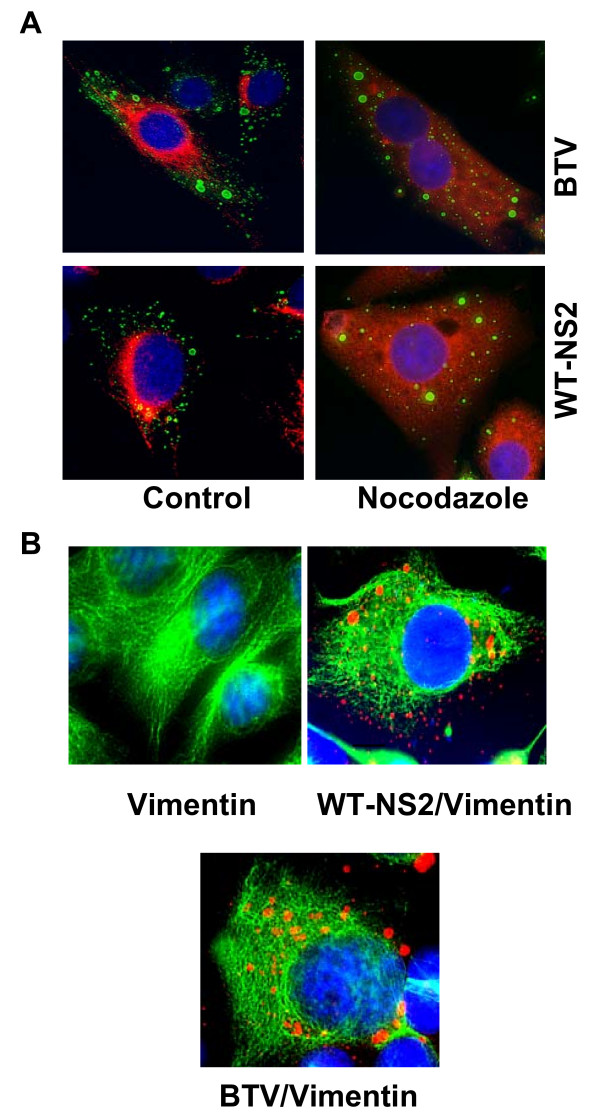
**NS2 IB does not associate with microtubule or vimentin**. (A) BSR cells infected with BTV-10 (upper panel) at 5 h p.i or modified BSR cells expressing NS2 (lower panel) were treated with nocodazole (right column) to disrupt microtubule. NS2 (green) and microtubule (red) were monitored. Untreated cells labeled for microtubule and NS2 (left column) were used as controls. (B) NS2 expressing cells (right) and BSR cells 24 h p.i were labeled for vimentin (green) and NS2 (red). Un-transfected and uninfected cells labeled for vimentin (left) were used as control.

A previous electron microscopy (EM) study has shown that during BTV infection virus-like particles and VIBs were associated with the intermediate filament component of cytoskeleton and not with intact microfilament or microtubule structures [27]. To investigate if the IBs produced by the stable cell lines behave in a similar manner we undertook an immunofluorescence study in the presence and absence of nocodazole, a microtubule depolymerizing drug. If IBs formation is dependent on tubulin, depolymerization should interfere in the formation and migration of IBs during the dynamic stage of production. For comparison we included BTV infected cells in parallel. We chose to treat the cells with drug at 5 and 15 h post-infection (p.i) since high density of VIBs are commonly visible in BTV infected cells between 12 and 16 h p.i [12]. From figure 2A it was evident that there was no obvious disruption or reorganization of IB formation between untreated cells or drug treated cells. The distribution of punctuate structures of NS2 IBs in NS2 expressing cells or VIBs in BTV infected cells were not affected by presence of nocodazole, although the ordered arrangement of microtubules compared to untreated cells was significantly disrupted (Fig 2A, upper and lower panels, right columns). Thus, the data indicate that NS2 IB formation is not dependent on cellular microtubules similar to that of BTV VIBs. This result also confirmed a previous finding from EM studies that microtubules were not involved in inclusion body morphogenesis [27].

For other members of the family a clear association between the virus particle, inclusions and the intermediate filament protein vimentin has been shown which was accompanied by significant changes in the vimentin filament network of the cells resulting in a sheath-like structure surrounding the inclusions [30]. To determine whether this association applied to NS2 IBs and if IBs in NS2 cells have similar effects on vimentin, NS2 expressing cells and BTV infected cells (control), were labeled with anti-vimentin monoclonal antibody (Fig. 2B, green) and anti-NS2 polyclonal antibody (Fig. 2B, red). The distribution of respective proteins was visualized by fluorescence microscopy. Unlike reoviruses, neither NS2 IBs nor BTV induced VIBs were surrounded by cage-like structures. This suggests that the filament network of cells is not involved in VIB formation and that NS2 IB, like VIB, does not require these filaments.

### The core proteins of BTV, but not the outer capsid protein VP2, is targeted to NS2 IBs

**Figure 3:**
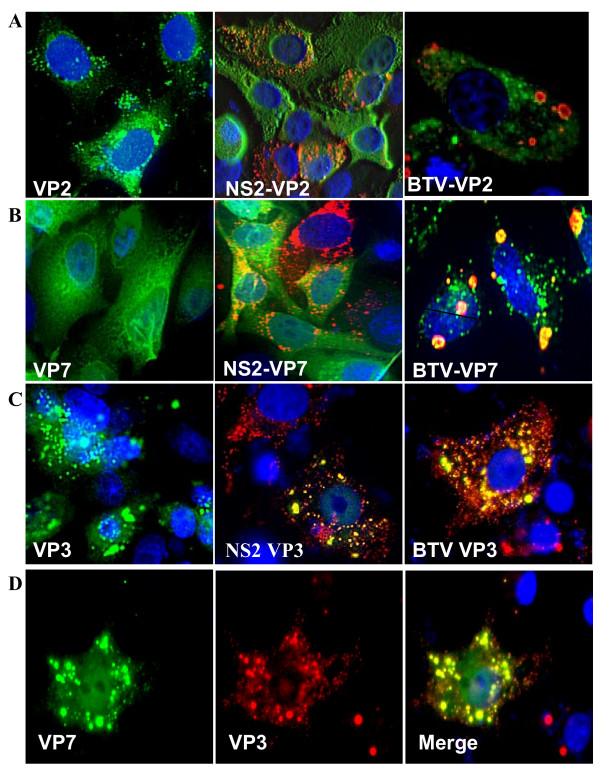
**Sub-cellular localization of BTV VP2, VP7 and VP3 in NS2 expressing or BTV infected BSR cells**. (A) Distribution of VP2 (green) and NS2 (red) in BSR cells transfected with plasmids expressing VP2 and NS2 (middle) or infected with BTV-10 (right). (B) Localization of VP7 (green) and NS2 (red) in transfected (middle) and infected cells (right). (C) Expression of VP3 (green) and NS2 (red) in transfected (middle) and infected cells (right). (D) Co-localization of VP7 (green) and VP3 (red) in full-length NS2 expressing BSR cells co-transfected with plasmids expressing VP7 and VP3. BSR cells transfected with VP2, VP7 and VP3 were used as controls (left column in A, B and C).

To investigate whether NS2 alone is sufficient to recruit the viral structural proteins into the IBs, we have monitored the localization of three major viral structural proteins; VP2 (outer capsid), VP7 (surface core protein) and VP3 (inner core protein that encapsidates the polymerase complex and viral genome). Each protein was expressed by transfecting plasmids into either control BSR cells (uninduced NS2 cells) or BSR cells expressing NS2 protein (induced NS2 cells). In addition, normal BSR cells infected by BTV were also used for comparison. The distribution of each protein was examined by immunofluorescence microscopy using anti-VP2, anti-VP3 or anti-VP7 antibody and inclusions were identified by using anti-NS2 antibody. When the distribution of NS2 and VP2 were monitored in NS2-BSR cells at 24 h post transfection by confocal microscopy (Fig. 3A, middle panel) no co-localization of VP2 and NS2 IBs could be detected and VP2 was distributed throughout the cytoplasm, similar to the control BSR cells (Fig. 3A, left panel). Further, when the distribution pattern of VP2 in NS2 expressing cells was compared with BTV infected cells (Fig. 3A, right panel), again no difference in VP2 distribution could be detected. Therefore the presence of other BTV components did not influence the VP2 intracellular localization.

In contrast, the distribution of VP7 in NS2 expressing cells was different to its distribution in BTV infected cells. Although VP7 was distributed throughout the cytoplasm in NS2 expressing cells similar to the control, uninduced BSR cells (Fig. 3B, left and middle panels), the distribution of VP7 was markedly different in BTV infected cells at 24 h p.i. All VIBs showed co-localization between NS2 and VP7, although there were also some foci of VP7 outside of VIBs (Fig. 3B, right panel). Evidently intracellular VP7 distribution is influenced by the presence of other BTV proteins.

In contrast to VP2 and VP7, the distribution of VP3 relative to IBs was strikingly different. In microscopy studies VP3 was exclusively localized within the VIBs in both NS2 expressing cells and in BTV infected cells (Fig. 3C, middle and right panels). The result was similar to that obtained recently with an EGFP tagged VP3 protein in BTV infected BSR cells [26]. These results are not surprising since VP3 is closely associated with the three core proteins (VP1, VP4 and VP6) that form the transcription complex and the BTV ssRNA. This complex subsequently generates the assembly intermediate, the single layered subcore particle which takes place within the VIBs. This data also confirmed our previous study that used transient expression of NS2 and core proteins [25]. Along with the previous publication, these data support the conclusion that unlike VP7, VP3 and inner transcription complex are closely associated with NS2 and VIBs. VP7 only associates with NS2 and VIBs through its interaction with VP3 or other BTV components. However, VP2 is never recruited within the VIBs.

### The distribution of VP7 in IBs alters in the presence of scaffolding protein VP3

To further our understanding of the influence of VP3 on VP7 assembly, we monitored the distribution pattern of both VP7 and VP3 in the presence of NS2 in BSR cells. NS2 expressing cells were co-transfected with plasmids expressing VP7 and VP3 and processed for immunostaining as described in the Materials and Methods. Both VP7 and VP3 were labeled with specific antibodies (Fig. 3D). The distribution of VP7 altered dramatically when co-expressed with VP3 in the NS2 expressing cells (Fig. 3D). Both VP3 and VP7 were perfectly co-localized in a pattern that closely resembled the pattern of NS2 IBs in the cytoplasm. The overall size of these IBs (VP3+VP7+NS2) was larger than that of NS2 IBs when NS2 was expressed singly or when BTV proteins were not associated with NS2 IBs (See Fig. 1 & Fig. 3A, middle column). It could be speculated that this is due to the presence of both VP3 and VP7 in NS2 IBs, thus increasing the overall sizes of NS2 IBs.

### RNA transcription takes place in NS2 IBs of a BTV infected cell

**Figure 4:**
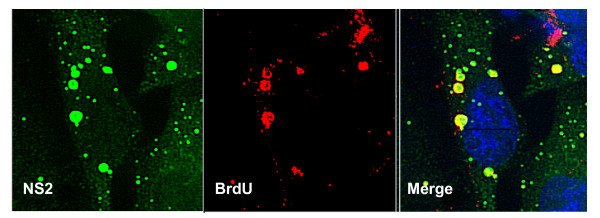
**Intracellular sites of RNA synthesis in BTV infected cells**. Co-localization of NS2 (green) and BrUTP labeled RNA (red) in BSR cells infected with BTV-10 (MOI of 1) 17 h p.i. The cells were treated with actinomycin D prior to immunostaining.

BTV positive strand RNA directs both protein synthesis and production of minus-strand RNA. In BTV infected cells the transcriptionally active core particles become rapidly surrounded by VIBs upon entry into the cytoplasm [27]. Since NS2 has an affinity for BTV transcripts [20] and is likely to be involved in the recruitment of viral transcripts, we sought to examine if the viral transcripts could be identified within the VIBs. BTV infected cells were treated with actinomycin D, to arrest the host replication and transcription, followed by transfection with BrUTP as described in Materials and Methods. We chose 14 h p.i for BrUTP transfection since the formation of VIBs in BTV infected cell was maximal between 12 and 16 h p.i. After fixation with formaldehyde newly made RNA was visualized by staining with anti-BrdU antibody and the NS2 inclusions were located using anti-NS2 polyclonal antibody. The BrdU-labeled RNA transcripts were distributed throughout the NS2 IBs suggesting that these are the likely sites for RNA replication (Fig. 4). BTV infected BSR cells in the absence of BrUTP were used as control and no BrdU signal was detected in these cells upon labeling with anti-BrUTP antibody, thus confirming its specificity (data not shown).

### Inducible expression of truncated NS2 derivatives in mammalian cells

**Figure 5:**
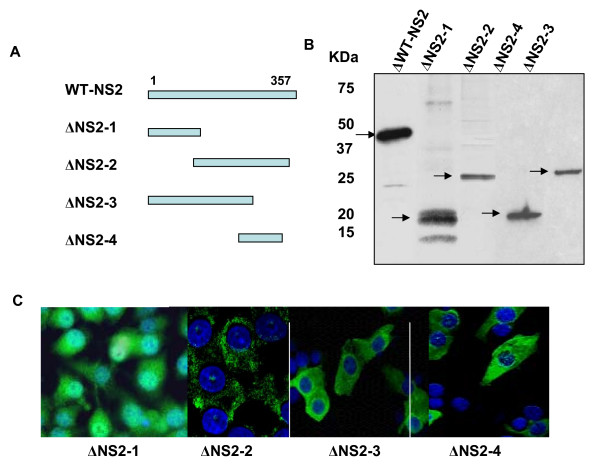
**Expression of NS2 mutants constitutively in BSR cells**. (A) Schematic representation of various NS2 carboxyl- and amino-terminal deletions used to produce inducible mammalian cell line (BSR). (B) Immunoprecipitation of radiolabeled proteins. Radiolabeled cell lysates of the different NS2 cell lines (BSR) were immunoprecipited using polyclonal anti-NS2 antibody. The names of the cell lines are given at the top of the panel. The positions of the various NS2 fragments are indicated. (C) Intracellular distribution of NS2 fragments (green) in inducible NS2 cell lines (BSR). The name of each mutant is indicated.

Upon confirmation of the fact that BTV core proteins, particles and transcripts were closely associated with NS2 and IBs formed by NS2, we extended our study to examine the role of NS2 in overall virus replication. To this end, we sought to obtain dominant negative mutants of NS2 in order to perturb the NS2 function. Deletion mutants of NS2 protein were designed in four overlapping fragments, residues 1–115 (ΔNS2-1), 116–357 (ΔNS2-2), 1–237 (ΔNS2-3), and 238–357 (ΔNS2-4) (Fig. 5A). BSR cell lines expressing each fragment constitutively were generated as described for the full-length NS2. The constitutive expression of each NS2 mutant fragment was determined by pulse labeling with ^35^S-methionine/cysteine and the labeled protein was immunoprecipitated with anti-NS2 BTV polyclonal antibody. The expression levels of each the truncated NS2 fragments when examined by SDS-PAGE was much lower than that of the full-length NS2 (Fig. 5B). In addition to the predicted molecular weight fragment of 1–115 (ΔNS2-1), an additional smaller breakdown product was also precipitated. To examine the capability of these NS2 fragments to produce IBs, cells expressing each NS2 truncation were labeled with the NS2 antibody and the distribution of IBs visualized by fluorescence microscopy. None of the truncated versions of NS2 could form the large IBs characteristic of BTV infection or like that of full-length NS2 (compare Fig. 1 with Fig. 5C). Instead the truncated NS2 proteins were found spread throughout the cell cytoplasm in a diffuse manner except ΔNS2-2, which exhibited some granular staining pattern (Fig. 5C). In all cases, the dispersed distribution of NS2 antigen completely disappeared in the presence of 1 μg/ml doxycycline indicating the absence of NS2 protein synthesis in the presence of the drug (data not shown). These results suggest that the capability to form IB is a function of the complete NS2 protein. Expression levels of the truncated NS2 proteins in ΔNS2-3 and ΔNS2-4 cell lines were found to be variable in the individual cells as indicated by the arrow (Fig. 5C). Due to this heterogeneity in protein expression these two cell lines were not used in subsequent experiments.

### Inhibitory effects of NS2 fragments on BTV infection

To investigate the effect of NS2 deletion mutants, cell line expressing full-length WT-NS2, ΔNS2-1 or ΔNS2-2 were infected with BTV and the level of infectious virus production in each case was monitored by plaque assay (in triplicates). The overall yield of infectious BTV particles, isolated from the culture media and the infected cell lysates of induced WT-NS2, was very similar to that of BTV infected normal BSR cells or uninduced cells (Table 1), as was the relative proportions of virus present in the cell-associated and supernatant fractions. However, when cell lines expressing the truncated NS2 were infected with BTV, both cell lines showed a reduced overall titre suggesting a trans-dominant inhibitory effect on virus replication. Moreover, in the case of ΔNS2-1, the overall titre was marginally reduced and the ratio of secreted to cell-associated virus titre increased about 1000 fold, while cell lines expressing ΔNS2-2 appeared to more powerfully inhibit overall virus replication.

**Figure 6:**
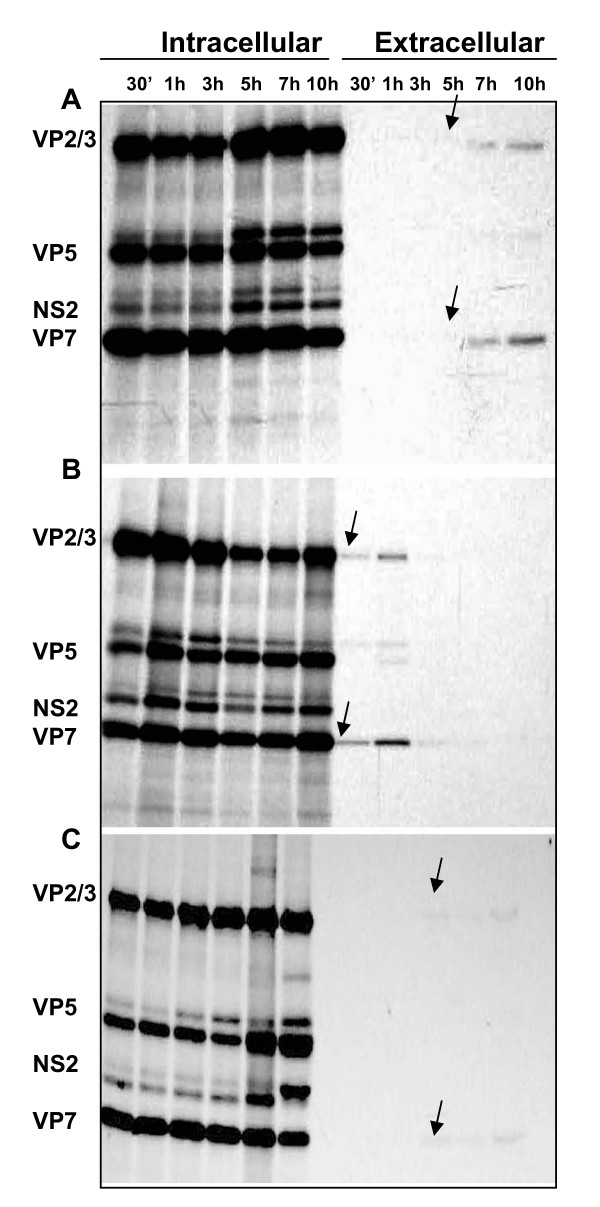
**Immunoprecipitation of the BTV proteins in NS2 cell lines**. Immunoprecipitation of BTV-10 proteins in NS2 cell lines; WT-NS2 (A), ΔNS2-1 (B) and ΔNS2-2 (C) were infected with BTV-10 and the lysates were precipitated by a polyclonal BTV antisera. The names of the fractions and post chase times are indicated at the top of the panel. Note the early virus release in ΔNS2-1 cells in compared to that of the other two cell lines as indicated by arrows.

To investigate this observation further, a pulse-chase analysis of each cell line was performed at 14 h p.i with BTV to assess the rate of virus movement through the cells. A 2 h labeling pulse was chased for 30 min, 1 h, 3 h, 5 h, 7 h and 10 h and at each time point both cell lysates and extra-cellular media were collected and BTV antigens identified by immunoprecipitation. Compared to the normal viral release as observed in WT-NS2 (Fig. 6 panel A), virus presence in the extra-cellular media of the ΔNS2-1 (aa1-115) cells was identifiable at very early time points (Fig. 6 panel B). The kinetics of virus release from ΔNS2-2 cells (panel C) resembled that of the full-length NS2 cell line (panel A), although the overall titre was considerably reduced (cf. panel A).

### Expression of only the RNA binding amino-terminal region of NS2 alters the BTV egress from the infected cell

**Figure 7:**
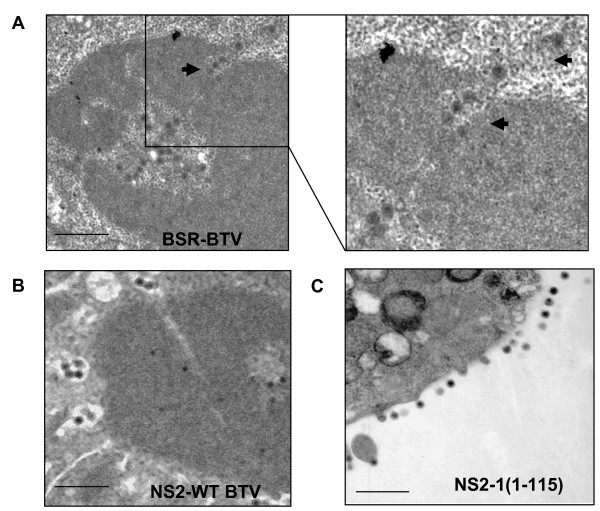
**Transmission electron micrographs illustrating VIBs and BTV distribution in BTV infected normal BSR and NS2 expressing cells**. (A) BTV-10 infected BSR cells, (B) full-length NS2 expressing cells and(C) ΔNS2-1 expressing cells were analyzed by transmission electron microscopy 24 h p.i. Normal infection of BTV particles are amplified (A right panel) to indicate the newly synthesized particles (indicated by arrows). Bar = 0.5 μm.

To examine further the effect of amino and carboxyl terminus truncation on IB formation and on BTV release, we examined each cell line following infection with BTV by EM. Negative staining of thin sections of BSR cells infected with BTV for 24 h prior to processing shows particulate structures with dimension ~70 nm (Fig. 7A). These structures were similar to BTV core particles and appeared to be surrounded by VIBs (Fig. 7A). Often these particles appeared to be released from the VIBs in a cluster (Fig. 7A right). Particles with similar sizes were also seen within the VIBs of the WT-NS2 cells that were infected with BTV (Fig. 7B). In contrast to the full-length NS2 expressing cells or normal BSR cells, cells expressing ΔNS2-1 showed release of particles resembling the BTV virion from the cell membrane, but very few, if any large VIBs (Fig. 7C). Thus, the normal virus egress pathway in mammalian cells was altered drastically due to the absence of IBs. Although in case of cells expressing ΔNS2-2, small granular bodies were observed, very few virus particles could be detected in the infected cells (data not shown).

## Discussion

The interaction of viral proteins and the recruitment of all the necessary components for *de novo *genomic RNA synthesis and viral core assembly have only recently begun to be illuminated. Reports from different laboratories, including this one, have shown that the different non structural proteins of diverse members of the *Reoviridae *family are involved in the formation of cytoplasmic inclusions that resemble viral IBs [17,31,32]. Despite the lack of sequence homology, functional analysis of rotavirus NSP2 (35 kDa) and reovirus protein σNS (41 kDa) suggests that they may be homologues of BTV NS2 as all three proteins have been implicated in viral genome replication and/or packaging [17,20,33]. Direct analysis of infected cells is limited by the fact that all viral proteins are constantly present and the distinction between proteins that are free in the cytoplasm or partially inclusion associated is difficult. In this study we created cell lines constitutively expressing BTV NS2 proteins, the main determinant of the viral inclusion formation, by using the Tet-Off inducible expression system, specifically to examine the association of NS2 with BTV structural proteins in the absence of the complete viral replication-cycle. It is known that BTV structural proteins have inherent affinities for each other as single, double or triple layered capsids can assemble in insect cells in the absence of NS2 or VIB formation [34,35]. However, in that system the concentration of structural proteins is very high and may obviate the need for facilitating molecules. A very similar phenomenon is observed with the assembly and release of retroviral capsids which proceeds following baculovirus expression without the P6 resident PTAP domain required for physiologically relevant assembly [36]. Thus, a plausible role for the VIBs in virus infected cells might be to coordinate the formation of infectious particles by the recruitment of all the essential structural components followed by the sequential packaging of viral genomes.

Inducible cells expressing NS2 were used to investigate which structural proteins associate with NS2 IBs in the absence of other BTV components. From this analysis it was clear that when the major BTV structural proteins were co-expressed singly with NS2, only the inner core protein VP3 co-localized with NS2 IBs. Neither VP7 nor VP2 protein was found to be associated with IB structures. However, when VP7 was expressed in the presence of VP3, VP7 was readily moved into the NS2 IBs. Thus, VP7 interacts spontaneously with VP3 to generate the core and assembly occurs predominantly at the site of VP3 accretion with NS2. The data also support our earlier findings which showed that VP3 and the three minor proteins that form the BTV transcription complex were always seen together with NS2 IBs when cells were transfected with NS2 plasmids or infected with BTV [25]. Together with our previous report and this current study we suggest that NS2 IBs recruit newly synthesized VP3 and the minor protein enzyme complex, soon after synthesis, in order to enucleate the essential components for virus replication. This model was further substantiated by demonstrating that the newly synthesized BTV transcripts were also localized within the NS2 IBs. This data is consistent with the data described recently which has shown that NS2 has specific binding affinity for BTV transcripts [20]. It seems therefore likely that NS2 IBs are the site where the co-assembly of VP3, the polymerase complex and plus strand BTV RNA species takes place. Studies of VP7 assembly onto the VP3 subcore have suggested that enshrouding begins at a number of discrete sites on the VP3 shell rather than one unique site [37]. Thus, either during subcore assembly or after completion of subcore formation, VP7 'T' trimers begin to attach to the VP3 subcore. The core particle subsequently forms a stable structure ready to release from the VIBs. During BTV infection subcore-like and core-like particles have been observed in IBs by scanning electron microscopy [5]. Neither outer virion structural protein, VP5 or VP2, have affinity for IBs and therefore progeny core particles produced in the VIBs most likely move to periphery of the VIB to be coated by the outer capsid proteins [12]. Interestingly, VP2 has been shown to be associated with the cell cytoskeleton [38]. The eukaryotic cytoskeleton network undergoes considerable rearrangement following infection by certain viruses such as African Swine Fever Virus (ASFV) [39], Vaccinia virus [40] and reovirus [30,41].

Some reovirus strains show selective and temporally regulated association of the cytoskeleton with the assembly site [42], and most mammalian reovirus strains form microtubule associated filamentous factories [31]. By contrast, visualization of BTV infected or NS2 expressing cells, by immunofluorescence, revealed that VIBs and NS2 IBs are globular cytoplasmic inclusions and are not associated with cytoskeleton. Most conclusively, the treatment of cells with a MT-depolymerizing agent, nocodazole, clearly indicated that there was no effect of the drug on VIBs formation in the cytoplasm.

To substantiate further the importance of NS2 and IBs in BTV replication, we generated cell lines expressing NS2 fragments. Two cell lines, one expressing amino terminal fragment and other carboxyl terminal fragment of NS2 fragments were utilized. The amino terminal 115 residues in NS2 have already been shown to contain the predominant RNA binding domain of the protein and thus may delineate functional boundaries within NS2 [20,28,29]. Neither fragment formed complete IBs alone but our data suggest that cells expressing the C-terminal fragment of NS2 (residues 116–357) were capable of limited NS2 antigen aggregation (punctuate as opposed to wholly diffuse staining in ΔNS2-1 cells). Protein-protein interactions via the C terminal portion of NS2 would be consistent with RNA interactions predominantly via the N-terminal 115 residues [28,29]. When the cell lines expressing WT-NS2 were infected with BTV, the constitutive expression of the complete molecule did not have a significant effect on virus yield nor on the distribution of virions between the cell and supernatant. However, BTV infection of the two cell lines expressing NS2 fragments lead to a marked reduction in overall virus yield possibly because both fragments act as dominant negatives through an interaction with the full-length virally expressed NS2. In addition, the relative proportion of virus released from the cell and the kinetics of release were altered. As revealed by pulse-chase and EM of infected cells, ΔNS2-1 cell line expressing the RNA binding region of NS2 severely crippled the ability of NS2 to form IBs and promoted accelerated trafficking of the progeny virus particles. It is known from our previous study that the interaction between RNA and NS2 plays a role in the stable oligomerisation of NS2 [25]. Hence, it can be hypothesized that perhaps ΔNS2-1 competes with the RNA binding activity of full-length NS2 and thereby perturbs the stability of VIBs which requires NS2-RNA interactions. In the case of ΔNS2-2, although the expression of small granular bodies could be observed, they were morphologically different to the functional inclusions formed by full-length NS2. Nevertheless, these small structures have the ability to interact with monomeric NS2 thereby interfering with the function of VIBs in virus replication. Thus, both mutants have the ability to perturb the normal virus replication and trafficking although the exact molecular basis behind these phenomena is not yet understood.

## Conclusion

The IBs or virosomes produced in BTV infected cells have been the subject of some debate, split broadly between them being integral to virus replication and assembly or being the inadvertent consequence of another primary function [20]. Our work shows that their previously described complexity (showing the presence of many structural and non-structural proteins) is a result of their key role in the recruitment of viral components. Their make-up is dependent only on a full-length NS2 with the C terminus of the protein being the major functional domain involved. NS2 has an innate propensity to interact with VP3, the inner core protein and through this has the ability to interact with VP7, the surface core protein. RNA binding by the amino terminal domain would complete the requirements for core formation and completed cores, now protected from the possibility of inducing an interferon response, would then be free to acquire the outer layers (VP5 and VP2) outside the IB structures. The data implies that replication and assembly are coordinated events in the IBs. The definition of the precise order of events required for completing BTV assembly and release, with the accompanying increased possibilities for prevention and control, remains an unfinished but achievable goal.

## Methods

### Virus and cells

BTV serotype 10 was plaque purified and propagated as described elsewhere [43]. BSR cells, a derivative of Baby hamster kidney cells (BHK), were maintained in Dulbecco's modified Eagle's medium (DMEM) supplemented with 10% fetal calf serum, penicillin and streptomycin. BSR Tet-Off cells were maintained as described above with the addition of antibody G418 sulphate to a final concentration of 100 μg/ml. BSR-NS2 cells were maintained in the same manner as BSR Tet-Off cells but with the addition of hygromycin to 100 μg/ml and doxycycline to 1 μg/ml.

### DNA manipulation and construction of DNA clones

Plasmid DNA manipulation was done essentially as described [44]. Restriction enzymes, T4 DNA ligase, DNA polymerase were purchased from New England Biolabs (NEB) and calf intestinal alkaline phosphatase was obtained from Roche (Germany).

### Construction of mutant NS2 genes and generation of inducible 'Tet Off' cell line

We used the following primers to clone NS2 mutants; forward primer 5' CGGGATCCCGAAATCCTTGAGTCATGGAGC 3' and reverse primer 5' CGGGATCCCG GTAAGTGTAAAATCCCCCC 3' for WT-NS2; forward primer 5' CGGGATCCCGAAATCCTTGAGTCATGGAGC 3' and reverse primer 5' CGGGATCCCGTTAAACCATCACACCATTATACTGTAC 3' for ΔNS2-1; forward primer 5' CGGGATCCCGATGGACGCTGAAATTAAATACTGTAAG 3' and reverse primer 5' CGGGATCCCGGTAAGTGTAAAATCCCCCC 3' for ΔNS2-2; forward primer 5' CGGGATCCCGAAATCCTTGAGTCATGGAGC 3' and reverse primer 5' CGGGATCCCGAAATCCTTGAGTCATGGAGC 3' for ΔNS2-3; forward primer 5' CGGGATCCCGATGTTTAAAGAGGTGGAAGC 3'and reverse primer 5' CGGGATCCCG GTAA GTGTAAAATCCCCCC 3' for ΔNS2-4. These *BamH *I cut PCR products were then ligated into the *Bam*H I site of pTRE2-Hyg (Clonetech) and used to transfect BSR Tet-Off mammalian cells (Clonetech). Stable cell lines expressing desired protein were selected using 200 μg/ml hygromycin and subsequently maintained with 100 μg/ml hygromycin.

### Labeling mammalian cells and immunoprecipitation

BSR cells expressing NS2 proteins in absence of doxycycline were incubated in medium lacking Methionine and Cysteine medium for 30 min and then pulsed with 100 μCi of ^35^S-Met/Cys for 2 h. The cells were harvested, washed and lysed with radio-immunoprecipitation (RIPA) buffer (50 mM Tris-HCl [pH 7.5], 150 mM NaCl, 1% Triton X-100, 0.5% sodium deoxycholate, 1 mM EDTA), incubated in ice for 15 min and centrifuged at 13,000 × g. Anti-NS2 polyclonal antiserum bound to protein A agarose beads (Pierce) was used to immunoprecipitate the complex form cell lysates. For immunoprcepitation of BTV proteins in NS2 cell lines, the cells were first infected with BTV 10 (MOI 0.1) and then processed for radiolabeling. Both intracellular and extracellular viruses were collected and subjected to immunoprecipitation with anti-BTV antibody. All the samples were washed with RIPA buffer, analyzed by SDS-PAGE and subjected to autoradiography. NS2 cell lines maintained in the presence of doxycycline and BSR cells were used as controls.

### Electron microscopy

NS2 expressing and BTV infected cells were pelleted at 2000 rpm for 10 min and fixed in 2.25% (w/v) glutaraldehyde 0.1 M cacodylate buffer (pH 7.2) at 4°C for 40 min. After rinsing the cells were post fixed in 1% (w/v) osmium tetroxide, buffered in 0.1 M cacodylate buffer (pH 7.2), washed in water, dehydrated in acetone and embedded in low viscosity resin. Sections were stained in 2% uranyl acetate. Stained sections were examined in a Hitachi H7000 electron microscope.

### Immunostaining and IF microscopy

For immunolabeling experiments 1.5 × 10^5 ^cells were seeded the day before infection or transfection in six-well plates (9.6 cm^2 ^per well) containing 18 mm round glass cover slips. Plasmids expressing VP2, VP3 or VP7 were transfected using lipofectamine 2000 (Invitrogen) according to manufacturer's recommendations. The cells were then processed for immunostaining as described previously [26]. For labeling VP2, the cells were incubated with anti-VP2 monoclonal antibody, while VP3, VP7 and NS2 were labeled with polyclonal antibodies followed by appropriate Alexa Fluor 488 (green)/FITC or Alexa Fluor 594 (red)/TRITC conjugated secondary antibodies. Immunostained cells were washed three times with PBS incubated with Hoechst 33342 for nuclear staining and mounted on slides with Vectashield (Vector laboratories). Immunolabeled samples were examined by Olympus inverted microscope with fluorescence optics. Images were collected digitally using IP lab software (Scananalytics, Fairfax, VA). Hoechst 33342, IgG conjugated to Alexa Fluor 488 or Alexa Fluor 594 were obtained from Molecular probes. Anti-β tubulin was purchased from Sigma Chemical Company. Anti-vimentin monoclonal was purchased from Oncogene.

### Detection of newly synthesized RNA *in vivo *by IF

BSR cells were infected with BTV at MOI of 1 for 14 h, followed by treatment with actinomycin D (10 μg/ml). After 1 h cells were transfected with 10 mM BrUTP (Sigma) using 5% FuGene transfection reagent (Roche). Cells were fixed 17 h pi and processed for IF analysis with mouse anti-bromodeoxyuridine BrdU monoclonal antisera (Sigma) followed by Alexa Fluor 594 goat anti-mouse IgG as secondary antibody.

### BTV infectivity assay

Induced NS2 cell lines were infected with BTV-10 at an MOI of 0.1 and incubated for 24 h at 37°C. Cell supernatant and pellets were collected and the cells were subjected to three freeze/thaw cycles to disrupt cells and release virus. After all the samples were collected, the viral of each sample was determined by virus plaque assay in BSR cell monolayer cultures. Uninduced NS2 cell lines and BSR cells were infected and processed as described above.

## Authors' contributions

AK carried out the molecular genetic studies, immunoprecipitation and plaque assays. BB maintained the cell lines and carried out confocal studies. PR conceived the study and participated in its design and coordination and helped to draft the manuscript. All authors read and approved the final manuscript.

## Figures and Tables

**Table 1:** Infectious BTV particles produced in NS2 expressing BSR cells

Cell Lines	Intracellular	Extracellular
**BSR/UNINDUCED**	5.0 × 10^7^	2.3 × 10^2^
**WT-NS2**	8.1 × 10^7^	4.8 × 10^2^
**ΔNS2-1**	7.1 × 10^6^	3.8 × 10^4^
**ΔNS2-2**	8.6 × 10^4^	8.2 × 10^2^

BSR cells, full-length NS2 cell lines (induced and uninduced) and two cell lines expressing NS2 fragments were infected with BTV-10 at MOI of 0.1. Released and intracellular virus titres at 24 h p.i were monitored by plaque assay. The values given here are an average of three different sets of plaque assay data.

## References

[B1] Eichwald C, Vascotto F, Fabbretti E, Burrone OR (2002). Rotavirus NSP5: mapping phosphorylation sites and kinase activation and viroplasm localization domains.. J Virol.

[B2] Touris-Otero F, Martinez-Costas J, Vakharia VN, Benavente J (2004). Avian reovirus nonstructural protein microNS forms viroplasm-like inclusions and recruits protein sigmaNS to these structures. Virology.

[B3] Szajner P, Jaffe H, Weisberg AS, Moss B (2003). Vaccinia virus G7L protein Interacts with the A30L protein and is required for association of viral membranes with dense viroplasm to form immature virions. J Virol.

[B4] Schwartz M, Chen J, Janda M, Sullivan M, den Boon J, Ahlquist P (2002). A positive-strand RNA virus replication complex parallels form and function of retrovirus capsids. Mol Cell.

[B5] Hyatt AD, Eaton BT (1988). Ultrastructural distribution of the major capsid proteins within bluetongue virus and infected cells. J Gen Virol.

[B6] Sadasiv EC, Chang PW, Gulka G (1985). Morphogenesis of canary poxvirus and its entrance into inclusion bodies. Am J Vet Res.

[B7] Forzan M, Wirblich C, Roy P (2004). A capsid protein of nonenveloped Bluetongue virus exhibits membrane fusion activity. Proc Natl Acad Sci USA.

[B8] Hassan SH, Roy P (1999). Expression and functional characterization of bluetongue virus VP2 protein: Role in cell entry. J Virol.

[B9] Hassan SH, Wirblich C, Forzan M, Roy P (2001). Expression and functional characterization of bluetongue virus VP5 protein: role in cellular permeabilization. J Virol.

[B10] Eaton BT, Hyatt AD, White JR (1988). Localization of the nonstructural protein NS1 in bluetongue virus-infected cells and its presence in virus particles. Virology.

[B11] Eaton BT, Gould AR (1987). Isolation and characterization of orbivirus genotypic variants. Virus Res.

[B12] Brookes SM, Hyatt AD, Eaton BT (1993). Characterization of virus inclusion bodies in bluetongue virus infected cells.. J Gen Virol.

[B13] Owens R, Roy P (2004). Role of an Arbovirus Nonstructural Protein in Cellular Pathogenesis and Virus Release.. J Virol.

[B14] Beaton AR, Rodriguez J, Reddy YK, Roy P (2002). The membrane trafficking protein calpactin forms a complex with bluetongue virus protein NS3 and mediates virus release. Proc Natl Acad Sci USA.

[B15] Huismans H, van Dijk AA, Bauskin AR (1987). In vitro phosphorylation and purification of a nonstructural protein of bluetongue virus with affinity for single-stranded RNA. J Virol.

[B16] Devaney MA, Kendall J, Grubman MJ (1988). Characterization of a non-structural phosphoprotein of two orbiviruses. Virus Res.

[B17] Thomas CP, Booth TF, Roy P (1990). Synthesis of bluetongue virus-encoded phosphoprotein and formation of inclusion bodies by recombinant baculovirus in insect cells: it binds the single-stranded RNA species. J Gen Virol.

[B18] Uitenweerde JM, Theron J, Stoltz MA, Huismans H (1995). The multimeric nonstructural NS2 proteins of bluetongue virus, African horsesickness virus, and epizootic hemorrhagic disease virus differ in their single-stranded RNA-binding ability. Virology.

[B19] Theron J, Nel LH (1997). Stable protein-RNA interaction involves the terminal domains of bluetongue virus mRNA, but not the terminally conserved sequences. Virology.

[B20] Lymperopoulos K, Wirblich C, Brierley I, Roy P (2003). Sequence specificity in the interaction of Bluetongue virus non-structural protein 2 (NS2) with viral RNA. J Biol Chem.

[B21] Markotter W, Theron J, Nel LH (2004). Segment specific inverted repeat sequences in bluetongue virus mRNA are required for interaction with the virus non structural protein NS2. Virus Res.

[B22] Butan C, Van Der Zandt H, Tucker PA (2004). Structure and assembly of the RNA binding domain of bluetongue virus non-structural protein 2. J Biol Chem.

[B23] Horscroft N, Roy P (2000). NTP-binding and phosphohydrolase activity associated with purified bluetongue virus non-structural protein NS2. Journal of General Virology.

[B24] Taraporewala Z, Chen D, Patton J (2001). Multimers of the bluetongue virus nonstructural protein, NS2, possess nucleotidyl phosphatase activity:similarities between NS2 and rotavirus NSP2.. Virology.

[B25] Modrof J, Lymperopoulos K, Roy P (2005). Phosphorylation of Bluetongue Virus Nonstructural Protein 2 Is Essential for Formation of Viral Inclusion Bodies. J Virol.

[B26] Kar AK, Iwatani N, Roy P (2005). Assembly and intracellular localization of the bluetongue virus core protein VP3. J Virol.

[B27] Eaton BT, Hyatt AD, White JR (1987). Association of bluetongue virus with the cytoskeleton. Virology.

[B28] Zhao Y, Thomas C, Bremer C, Roy P (1994). Deletion and mutational analyses of bluetongue virus NS2 protein indicate that the amino but not the carboxy terminus of the protein is critical for RNA-protein interactions. J Virol.

[B29] Fillmore GC, Lin H, Li JKK (2002). Localization of the single-stranded RNA-binding domains of bluetongue virus nonstructural protein NS2.. J Virol.

[B30] Sharpe AH, Chen LB, Fields BN (1982). The interaction of mammalian reoviruses with the cytoskeleton of monkey kidney CV-1 cells. Virology.

[B31] Broering TJ, Parker JS, Joyce PL, Kim J, Nibert ML (2002). Mammalian reovirus nonstructural protein muNS forms large inclusions and colocalizes with reovirus microtubule-associated protein mu2 in transfected cells. J Virol.

[B32] Mohan KV, Muller J, Som I, Atreya CD (2003). The N- and C-terminal regions of rotavirus NSP5 are the critical determinants for the formation of viroplasm-like structures independent of NSP2. J Virol.

[B33] Gillian A, Nibert M (1998). Amino terminus of reovirus nonstructural protein sigma NS is important for ssRNA binding and nucleoprotein complex formation.. Virology.

[B34] French TJ, Roy P (1990). Synthesis of bluetongue virus (BTV) corelike particles by a recombinant baculovirus expressing the two major structural core proteins of BTV. J Virol.

[B35] French TJ, Marshall JJ, Roy P (1990). Assembly of double-shelled, virus-like particles of bluetongue virus by the simultaneous expression of four structural proteins. J Virol.

[B36] Adamson CS, Jones IM (2004). The molecular basis of HIV capsid assembly--five years of progress. Rev Med Virol.

[B37] Limn CK, Roy P (2003). Intermolecular interactions in a two-layered viral capsid that requires a complex symmetry mismatch. J Virol.

[B38] Bhattacharya B, Noad R, P. R (2007). Interaction between Bluetongue virus outer capsid protein VP2 and vimentin is necessary for virus egress. Virology Journal.

[B39] Heath CM, Windsor M, Wileman T (2001). Aggresomes resemble sites specialized for virus assembly. J Cell Biol.

[B40] Risco C, Rodriguez JR, Lopez-Iglesias C, Carrascosa JL, Esteban M, Rodriguez D (2002). Endoplasmic reticulum-Golgi intermediate compartment membranes and vimentin filaments participate in vaccinia virus assembly. J Virol.

[B41] Greber UF, Way M (2006). A superhighway to virus infection. Cell.

[B42] Mora M, Partin K, Bhatia M, Partin J, Carter C (1987). Association of reovirus proteins with the structural matrix of infected cells. Virology.

[B43] Mertens PP, Burroughs JN, Anderson J (1987). Purification and properties of virus particles, infectious subviral particles, and cores of bluetongue virus serotypes 1 and 4. Virology.

[B44] Sambrook J, Russell DW (2001). Molecular Cloning: A Laboratory Manual.

